# Diagnostic discordance between referral impressions, clinical assessment, and final electrodiagnostic findings

**DOI:** 10.1016/j.cnp.2026.09.008

**Published:** 2026-10-12

**Authors:** Lauren L. Walkon, Daniel Menkes

**Affiliations:** Oakland University William Beaumont School of Medicine, Auburn Hills, MI, USA

**Keywords:** Electromyography, Nerve conduction studies, Diagnostic concordance, Neuromuscular disorders, Clinical decision-making, Peripheral neuropathy, Electrodiagnosis

## Abstract

**Objective:**

This study evaluated concordance between referral impressions, structured pre-test assessment, and electrodiagnostic findings (EDX), compared the concordance of an electrodiagnostic consultant's (EC) pre-test assessment and the referring clinician's impression with the final EDX diagnosis.

**Methods:**

A single-center, prospective observational study enrolled 104 participants over 9 months; 101 completed testing. The EC recorded a diagnostic impression before nerve conduction studies and needle electromyography (EMG). Final diagnoses were categorized using an ICD-10 root-based schema. Referral, pre-test, and final diagnoses were compared using exact agreement between the first-listed primary ICD-10 diagnosis codes.

**Results:**

Among 101 patients (mean age 59 years; 59.4% female), referral-final concordance was 34.7% (35/101), compared with 71.3% (72/101) for EC pre-test-final concordance (matched OR, 6.29; 95% CI, 2.81–16.54; *p* < 0.001). EDX produced a clinically meaningful diagnostic change in 67 patients (66.3%) and modified management in 53 (52.5%); subsequent implementation was not assessed. The EC pre-test diagnosis differed from the final EDX diagnosis in 29 cases (28.7%).

**Conclusions:**

EDX frequently identified diagnoses that differed from referral impressions and sometimes differed from the consultant's pre-test assessment. The higher pre-test-final concordance reflects within-clinician agreement after a focused history and examination; it does not establish that the electrodiagnostic consultant is more diagnostically accurate than referring clinicians.

**Significance:**

This single-center exploratory study supports the clinical value of a focused pre-test assessment and of EDX as a diagnostic consultation. Larger, multicenter studies using independent assessment are needed.

## Highlights

• Electrodiagnostic testing changed the diagnosis in 66.3% of patients.
• Referring diagnoses matched final findings in only 34.7% of patients.
• An electromyographer's pretest assessment matched final findings in 71.3%.

## 1 Introduction

Electrodiagnostic evaluation is most appropriately understood as a diagnostic consultation that integrates the referral question, a focused neuromuscular history and examination, nerve conduction studies, and needle EMG. Prior studies have documented variable concordance between referral diagnoses and EDX findings, as well as clinically important new or alternative diagnoses after testing. However, the literature includes heterogeneous referral populations, frequently relies on retrospective designs, and has limited prospective data describing the relation between a structured pre-test assessment and final EDX findings or the downstream management implications of the EDX report.

Several retrospective studies have evaluated EMG diagnostic patterns and reported findings suggesting substantial discordance between referring diagnoses and subsequent electrodiagnostic findings. More recent studies reported an overall concordance between referring diagnosis and EMG findings ranging from 37% to 79.9% (Haig et al., 1999; Kothari et al., 1998). Another retrospective study reported that 61.8% of clinical diagnoses were confirmed through electrodiagnostic studies (López-López et al., 2025). Bhadola and Daniello reported that only 50.8% of referring diagnoses were confirmed in a retrospective review of 602 patients, with particularly low confirmation rates for carpal tunnel syndrome referrals (Bhadola and Daniello, 2017). A large prospective study found that EMG confirmed the referral diagnosis in 61.9% of cases while providing new clinically significant diagnoses in 32.4% of patients (Bembenek et al., 2023).

A recurrent finding across these multiple studies is the high rate of normal EMG results, ranging from 25.1% to 55% of all referrals, suggesting significant overutilization of EMG testing (Bembenek et al., 2023). However, when abnormalities are found, EMG has provided substantial diagnostic value, with studies reporting new or unsuspected diagnoses in 16% to 42% of cases (Nikolić et al., 2016). Notably, So et al. reported that electrodiagnostic testing provides new or unsuspected diagnoses in 31–43% of cases (So, 2009).

Another key consideration is variation in concordance rates across specialties. The literature reports that neuromuscular neurologists achieve higher concordance rates than general neurologists, and specialists achieve 77% concordance with the EMG result as compared to 55% for general practitioners (Cocito et al., 2006; Nikolić et al., 2016). Neurology departments demonstrated the highest concordance (κ = 0.853), while neurosurgery had the lowest (κ = 0.502) López-López et al., 2025).

The literature also identifies specific clinical factors that predict EMG utility. Pain and weakness were significantly associated with diagnostic confirmation, with odds ratios of 3.1 and 2.46, respectively (López-López et al., 2025). In contrast, pain as an isolated symptom resulted in normal studies 41.2% of the time (Bhadola and Daniello, 2018). Multivariate analysis showed that paresthesia, weakness, and sensory loss on examination positively predicted abnormal findings, while pain had a negative correlation (Podnar, 2005).

A critical finding is that only 32.1% of referrals included physical examination findings, and no patient with a normal clinical examination had an abnormal EMG result except for carpal tunnel syndrome (López-López et al., 2025; Podnar, 2005). This highlights the importance of an adequate clinical evaluation before requesting an EMG.

This prospective single-center exploratory investigation was designed to describe three related, but distinct, forms of agreement: referral-final EDX concordance, electrodiagnostic consultant pre-test-final EDX concordance, and diagnostic reclassification after EDX. The latter comparison should be interpreted as within-clinician agreement, because the same electrodiagnostic consultant performed the focused assessment, selected the study protocol, and interpreted the final EDX findings. It therefore does not provide an unbiased comparison of diagnostic accuracy between the electrodiagnostic consultant and the referring clinician.

The study's contribution is the prospective, structured capture of a pre-test clinical impression before EDX and the descriptive evaluation of recorded management-plan modifications after final EDX reporting. We additionally examine patterns by principal referral indication, recognizing that EDX yield and clinical utility differ across entrapment neuropathies, polyneuropathy, radiculopathy, pain-based referrals, and sensory-based referrals. These analyses are intended to be descriptive and hypothesis-generating rather than definitive comparative effectiveness estimates.

### 1.1 Study significance

This prospective single-center exploratory investigation was designed to describe three related but distinct outcomes: referral-final diagnostic agreement, electrodiagnostic consultant pre-test-final diagnostic agreement, and referral-based diagnostic reclassification after EDX. The consultant pre-test-final comparison is a measure of within-clinician agreement after a focused history and examination. Because the same consultant selected the EDX protocol and interpreted the final EDX findings, it is not an unbiased measure of comparative diagnostic accuracy versus the referring clinician.

The study prospectively captured a pre-test clinical impression before EDX and a recorded management-plan modification outcome after final EDX reporting. It additionally describes patterns by principal referral indication, recognizing that EDX yield may differ among entrapment neuropathy, polyneuropathy, radiculopathy, pain-based, and sensory-based referrals. These subgroup analyses are descriptive and hypothesis-generating.

## 2 Methods

### 2.1 Study design and setting

This prospective observational study was conducted at ***** with local Institutional Review Board approval. Verbal informed consent was obtained from participants for the anonymous use of their clinical data. Participant information was obtained over nine months from March 2025 to December 2025.

### 2.2 Study workflow

The study workflow followed a sequential, pre-specified order designed to minimize bias. Upon patient arrival, the electrodiagnostic consultant (EC) reviewed the electronic referral, including the referring provider's diagnosis and clinical question. Next, the EC obtained a directed history and performed a focused neurological examination. Before nerve conduction studies or needle electromyography was performed, the EC recorded a primary pre-test diagnostic impression on a standardized data-collection form. After completion of the EDX evaluation, the EC interpreted the findings and recorded a primary final diagnostic impression. For analysis, the referral diagnosis, EC pre-test impression, and final EDX impression were assigned primary ICD-10 codes. The EC prospectively recorded the first-listed ICD-10 code for the referral diagnosis, pre-test impression, and final EDX diagnosis on the standardized data-collection form. Primary-code concordance was determined by exact agreement between the first-listed ICD-10 diagnosis codes at the two stages being compared. Codes were not collapsed to their broader root categories for the primary concordance analyses.

The EC/electromyographer performed the outcome classification, including assignment of the primary diagnostic category, referral confirmation, diagnostic change, and recorded management-plan modification. The same EC conducted the focused pre-test assessment, selected the EDX protocol, performed the needle examination, interpreted the final EDX findings, and classified the study outcomes. These classifications were not independently adjudicated or performed under blinded conditions. This design enabled prospective and internally consistent data collection but introduced incorporation, observer, and expectation bias, particularly in comparisons between the EC's pre-test and final EDX impressions.

### 2.3 Participants and inclusion/exclusion criteria

Data were collected during patient encounters at their scheduled EMG visits. To ensure consistency in evaluation, all patient encounters were assessed by the same physician who also performed the EMG.

Inclusion criteria were: (1) age ≥ 13 years, (2) ability to give informed consent (by patient or legal guardian), (3) willingness to provide verbal consent for anonymous data use, and (4) completion of the scheduled EDX study.

### 2.4 Statistical analysis

Descriptive statistics are reported as mean ± standard deviation or number and percentage. Exact binomial 95% confidence intervals were calculated for key proportions. Referral-final and consultant pre-test-final concordance were assessed in the same patients and compared using an exact McNemar test. The matched odds ratio was calculated from discordant pairs, with an exact conditional 95% confidence interval. Associations between binary diagnostic and management outcomes were evaluated using Fisher exact tests and odds ratios with 95% confidence intervals. Disease-specific analyses were descriptive because of small, uneven subgroup sizes. All primary analyses were two-sided with an alpha threshold set at α = 0.05. There was no missing data for any of the analyzed variables.

Indication-specific primary-code concordance was calculated within each principal referral group using the same exact first-listed ICD-10 code definition as the overall analysis. Referral-final concordance represented agreement between the primary referral code and the primary final EDX code. EC pre-test–final concordance represented agreement between the EC's primary diagnosis after the focused history and examination—but before testing—and the primary final EDX code. Because referral groups were small and uneven, indication-specific rates were summarized descriptively as numerators and percentages; no formal between-group hypothesis tests were performed.

### 2.5 Participant flow

A total of 104 participants were enrolled during the nine-month study period. Three participants did not complete the scheduled EDX evaluation and were excluded in accordance with the eligibility criteria. The final analytic cohort therefore included 101 participants who completed testing. All analyzed variables were available for these 101 participants.

### 2.6 Electromyography protocol and equipment

All nerve conduction studies were performed by a single board-certified electrodiagnostic technologist using a Cadwell Sierra Summit EMG system, whereas needle EMG studies were performed by a board-certified EC. Standardized techniques followed the American Association of Neuromuscular & Electrodiagnostic Medicine (AANEM) guidelines. Nerve conduction studies were performed using surface electrodes with skin temperature maintained at 30–32C in the lower extremities and ≥ 32 °C and < 34 °C in the upper extremities. Sensory nerve conduction studies were performed antidromically. Motor nerve conduction studies included distal latency, amplitude, conduction velocity, and F-wave assessments as clinically indicated. Needle electromyography was performed using a 37-mm concentric needle electrode and assessed spontaneous activity, motor-unit morphology, and recruitment. The number and distribution of muscles examined were individualized to the referral question; approximately 5–20 muscles were sampled per patient, not per limb. Symptomatic and contralateral limbs were evaluated when clinically appropriate.

### 2.7 Outcome measures and diagnostic classification

Three related but distinct diagnostic outcomes were evaluated: primary-code concordance, referral confirmation, and diagnostic change.

Primary-code concordance was defined as an exact match between the first-listed primary ICD-10 diagnosis codes at the two stages being compared. Referral-final concordance compared the first-listed referral diagnosis with the first-listed final EDX diagnosis. EC pre-test–final concordance compared the EC's first-listed pre-test diagnosis, recorded after the focused history and examination but before testing, with the first-listed final EDX diagnosis. Secondary diagnoses were not considered when determining primary-code concordance. All classifications were performed by the EC/electromyographer. Referral groups were assigned according to the first-listed referral code and were collapsed into the prespecified categories shown in Table 3.

Referral confirmation was coded separately as a binary clinical-classification outcome. A referral diagnosis was considered confirmed when the final EDX findings supported the principal condition or localization suspected by the referring clinician, even if the first-listed final ICD-10 code was not identical to the first-listed referral code. Thus, referral confirmation allowed clinically supportive findings that did not satisfy the stricter primary-code concordance definition.

Diagnostic change was also coded as a separate binary outcome. A clinically meaningful diagnostic change was considered present when the final EDX evaluation materially revised the principal referral impression by identifying an alternative diagnosis or localization, adding a clinically consequential diagnosis, or demonstrating a normal/non-localizing result that changed the interpretation of the presenting problem. Minor wording differences or changes that did not alter the clinical interpretation were not classified as diagnostic changes.

Recorded Management-Plan Modifications: Immediately after completion and interpretation of the EDX evaluation, the EC recorded whether the final EDX findings resulted in a clinically meaningful management recommendation or intended plan modification. This variable represents a management recommendation or intended plan recorded by the EC immediately after the assessment. It does not establish that the referring clinician accepted the recommendation, that the proposed treatment or investigation was subsequently ordered, that the patient initiated or completed it, or that it improved patient outcomes. The referring clinician retained responsibility for final management decisions. The study did not conduct longitudinal follow-up to confirm implementation.

The EC/electromyographer assigned the diagnostic codes and classified referral confirmation, diagnostic change, and recorded management-plan modification. The EC/electromyographer prospectively assigned the diagnostic codes and classified referral confirmation, diagnostic change, and recorded management-plan modification. Primary-code concordance was subsequently calculated from the first-listed ICD-10 codes. Outcome classifications were not independently adjudicated. Primary-code concordance, referral confirmation, and diagnostic change were analyzed as distinct and noninterchangeable outcomes.

## 3 Results

### 3.1 Patient demographics and clinical characteristics

A total of 101 patients completed the study (mean age 59 ± 18 years, 59.4% female). The cohort included patients with diabetes (14.9%) and prior neuropathy history (16.8%) (Table 1).

**Table 1:** Participant characteristics.

Characteristic	Value
Participants analyzed	101
Age, years, mean ± SD	59 ± 18
Female sex, n (%)	60 (59.4%)
Diabetes, n (%)	15 (14.9%)
Prior neuropathy history, n (%)	17 (16.8%)

### 3.2 Diagnostic agreement and paired analysis

Exact raw-text agreement between the consultant's pre-test impression and final EDX diagnosis was observed in 68 of 101 cases (67.3%). Agreement on the primary listed ICD-10 diagnosis code was observed in 72 cases (71.3%; 95% CI, 61.4%–79.9%), while the consultant's primary pre-test diagnosis differed from the final EDX diagnosis in 29 cases (28.7%; 95% CI, 20.1%–38.6%). Referral-final primary-code concordance was observed in 35 cases (34.7%).

These primary-code concordance outcomes were distinct from the separately classified referral-confirmation and diagnostic-change outcomes. Primary-code concordance required exact agreement between the first-listed ICD-10 diagnosis codes, whereas referral confirmation allowed clinically supportive findings that did not necessarily share the same primary ICD-10 code. Diagnostic change captured clinically meaningful reclassification rather than simple code disagreement.

In paired analysis, 44 cases were concordant only for the consultant pre-test impression, 7 were concordant only for the referral diagnosis, 28 were concordant for both assessments, and 22 were discordant for both assessments (Table 2). Consultant pre-test-final concordance was more frequent than referral-final concordance (exact McNemar *p* < 0.001; matched OR, 6.29; exact conditional 95% CI, 2.81–16.54). The absolute paired difference in concordance was 36.6 percentage points (approximate paired 95% CI, 24.8–48.5 percentage points).

**Table 2:** Paired diagnostic agreement using the primary listed ICD-10 diagnosis code.

Referral-final concordance	EC pre-test-final discordant	EC pre-test-final concordant	Total
Referral-final discordant	22	44	66
Referral-final concordant	7	28	35
Total	29	72	101

Concordance required an exact match between the first-listed primary ICD-10 diagnosis codes at the compared stages. Secondary diagnoses were not considered when determining primary-code concordance. Exact McNemar *p* < 0.001. Matched odds ratio for EC versus referral concordance, 6.29; exact conditional 95% CI, 2.81–16.54. EC, electrodiagnostic consultant; EDX, electrodiagnostic evaluation; ICD-10, International Classification of Diseases, Tenth Revision.

### 3.3 Recorded management-plan modifications

Immediately after completion and interpretation of the EDX evaluation, the EC recorded a management recommendation or intended plan modification in 53 of 101 patients (52.5%; 95% CI, 42.3%–62.5%). This outcome reflects the plan documented by the EC at the conclusion of the assessment and does not represent independently verified treatment implementation, completion of a recommended investigation or referral, or improvement in clinical outcomes.

A recorded management-plan modification was more frequent among patients whose referral diagnosis was not classified as confirmed than among those whose referral diagnosis was classified as confirmed: 38 of 61 patients (62.3%) versus 15 of 40 patients (37.5%), respectively (OR, 2.75; 95% CI, 1.21–6.27; Fisher exact *p* = 0.024). Modifications were also more frequent among patients with a coded diagnostic change than among those without, 41 of 67 patients (61.2%) versus 12 of 34 patients (35.3%), respectively (OR, 2.89; 95% CI, 1.23–6.82; Fisher exact *p* = 0.020).

### 3.4 Specialty-specific patterns

The analytic dataset included 53 primary care/generalist referrals, 23 neurology referrals, 21 musculoskeletal/orthopedic referrals, and 4 referrals from other specialties. Referral-final primary-code concordance was observed in 14 of 53 primary care/generalist referrals (26.4%), 9 of 23 neurology referrals (39.1%), 11 of 21 musculoskeletal/orthopedic referrals (52.4%), and 1 of 4 referrals from other specialties (25.0%). Consultant pre-test-final primary-code concordance was 71.7%, 56.5%, 81.0%, and 100.0%, respectively.

Because of sparse cells, particularly in the other-specialty group, specialty-specific findings are presented descriptively and should not be interpreted as definitive comparative results.

### 3.5 EDX outcomes by referral indication

Indication-specific concordance rates are shown in Table 3. Referral-final primary-code concordance was highest among entrapment-neuropathy referrals, occurring in 18 of 23 cases (78.3%), followed by sensory-based referrals in 10 of 16 cases (62.5%) and polyneuropathy referrals in 7 of 17 cases (41.2%). No exact primary-code concordance was observed among the radiculopathy, pain-based, or other referral groups. EC pre-test–final concordance was highest among radiculopathy referrals, occurring in 15 of 18 cases (83.3%), followed by entrapment-neuropathy referrals in 19 of 23 cases (82.6%), polyneuropathy referrals in 13 of 17 cases (76.5%), other referrals in 12 of 20 cases (60.0%), pain-based referrals in 4 of 7 cases (57.1%), and sensory-based referrals in 9 of 16 cases (56.3%). These indication-specific findings are descriptive because of the small and uneven subgroup sizes.

**Table 3:** Final EDX patterns and primary-code concordance by principal referral indication.

Principal referral indication	N	Normal/non-localizing, n (%)	Specific final EDX diagnosis, n (%)	Primary final EDX roots, n	Referral-final concordance, n (%)	EC pre-test–final concordance, n (%)	No diagnostic change, n (%)	Management modification, n (%)
Entrapment neuropathy (G56/G57)	23	1 (4.3%)	22 (95.7%)	G56 (21); G57 (1)	18 (78.3%)	19 (82.6%)	11 (47.8%)	22 (95.7%)
Polyneuropathy (G62/E11.4)	17	3 (17.6%)	14 (82.4%)	G62 (7); G56 (3); M54 (2); E11 (2)	7 (41.2%)	13 (76.5%)	11 (64.7%)	5 (29.4%)
Radiculopathy (M54.1)	18	5 (27.8%)	13 (72.2%)	G56 (8); M76 (1); G57 (1); M79 (1); M54 (1); G62 (1)	0 (0.0%)	15 (83.3%)	1 (5.6%)	7 (38.9%)
Pain-based (M79.x)	7	2 (28.6%)	5 (71.4%)	G56 (3); G62 (2)	0 (0.0%)	4 (57.1%)	1 (14.3%)	4 (57.1%)
Sensory-based (R20.x)	16	10 (62.5%)	6 (37.5%)	G56 (5); M62 (1)	10 (62.5%)	9 (56.3%)	7 (43.8%)	6 (37.5%)
Other	20	5 (25.0%)	15 (75.0%)	G56 (5); M54 (2); G54 (2); G62 (1); P14 (1); M62 (1); G70 (1); Q66 (1); M25 (1)	0 (0.0%)	12 (60.0%)	3 (15.0%)	9 (45.0%)
Total	101	26 (25.7%)	75 (74.3%)	–	35 (34.7%)	72 (71.3%)	34 (33.7%)	53 (52.5%)

EDX, electrodiagnostic evaluation; EC, electrodiagnostic consultant; ICD-10, International Classification of Diseases, Tenth Revision. Principal referral groups were assigned according to the first-listed referral diagnosis. Primary-code concordance required an exact match between the first-listed primary ICD-10 codes at the relevant stages; agreement at a broader root-category level without an exact primary-code match was not classified as concordant. Referral-final concordance compared the first-listed referral and final EDX codes. EC pre-test–final concordance compared the EC's first-listed pre-test diagnosis with the first-listed final EDX diagnosis.

Normal/non-localizing findings included a normal EDX evaluation or an isolated R20 diagnosis without a specific electrophysiologically localizing diagnosis. Accordingly, the isolated R20 diagnosis in the sensory-based group was classified as normal/non-localizing and excluded from the specific-diagnosis column. Management modification represents a recommendation or intended plan recorded by the EC immediately after completion and interpretation of the EDX evaluation; subsequent implementation was not verified. Subgroup findings are descriptive, and no formal comparisons among referral-indication groups were performed.

ICD-10 categories: E11.4, type 2 diabetes mellitus with neurologic complications; G54, nerve root and plexus disorders; G56, mononeuropathies of the upper limb; G57, mononeuropathies of the lower limb; G62, other and unspecified polyneuropathies; G70, myasthenia gravis and other neuromuscular junction disorders; M25, other joint disorders; M54, dorsalgia, including radiculopathy; M62, other muscle disorders; M76, enthesopathies of the lower limb, excluding the foot; M79, other soft-tissue disorders, including limb pain; P14, birth injury to the peripheral nervous system; Q66, congenital deformities of the feet; and R20, disturbances of skin sensation.

After correction of the classification of one isolated R20 diagnosis, 10 of 16 sensory-based referrals (62.5%) had normal or non-localizing final findings and 6 of 16 (37.5%) had a specific final EDX diagnosis. The specific diagnoses in this group consisted of five upper-limb mononeuropathies and one muscle-disorder diagnosis.

## 4 Discussion

In this prospective single-center exploratory investigation, agreement between the electrodiagnostic consultant's focused pre-test impression and final EDX primary diagnosis was more frequent than agreement between the referral diagnosis and final EDX primary diagnosis. The pre-test-final comparison is a within-clinician measure because the same consultant obtained the history, performed the examination, selected the testing protocol, and interpreted the final EDX findings. It should therefore be viewed as evidence that a focused pre-test assessment is informative in this setting, not as proof that electrodiagnostic consultants are inherently more accurate diagnosticians than referring clinicians.

Final EDX differed from the consultant's primary pre-test diagnosis in 28.7% of patients. This residual discordance represents the incremental diagnostic contribution of EDX after a focused assessment and is consistent with the clinical role of nerve conduction studies and needle EMG in confirming, localizing, characterizing, or revising a suspected diagnosis. The present study did not evaluate patient-reported outcomes, recovery, prognostication, surgical decision accuracy, or long-term clinical outcomes.

The paired comparison was evaluated with an exact McNemar test rather than a test for independent proportions. Forty-four patients were concordant only for the consultant pre-test assessment and seven only for the referral impression, producing a matched odds ratio of 6.29. Although this test accounts for the paired data structure, it does not eliminate the incorporation and observer bias intrinsic to the within-clinician comparison.

Disease-specific patterns were clinically informative but should be interpreted cautiously. Suspected entrapment neuropathy commonly resulted in a specific final EDX diagnosis, whereas sensory-based referrals more often resulted in normal or non-localizing findings. After correction of the classification of an isolated R20 diagnosis, 10 of 16 sensory-based referrals had normal or non-localizing findings and 6 had a specific final diagnosis. Thus, although isolated sensory symptoms frequently lacked an electrophysiologically localizing correlate, a substantial minority were associated with a specific diagnosis, most commonly an upper-limb mononeuropathy.

Recent studies in *Clinical Neurophysiology Practice* further demonstrate that the diagnostic contribution of nerve conduction studies depends on the clinical population, protocol, and electrophysiologic measures selected. Sand et al. used a comprehensive NCS protocol to evaluate long-term peripheral nerve findings after Roux-en-Y gastric bypass and demonstrated how a comprehensive NCS protocol could characterize peripheral nerve abnormalities, including polyneuropathy and carpal tunnel syndrome, in a defined clinical population. Yano et al. reported that the tibial-to-ulnar compound muscle action potential amplitude ratio could help characterize length-dependent neuropathy and distinguish acquired from inherited neuropathy phenotypes. Together, these studies emphasize that NCS findings should be interpreted within an appropriate clinical and methodologic framework and that quantitative electrophysiologic measures may provide diagnostic information beyond a binary normal-versus-abnormal classification (Sand et al., 2024; Yano et al., 2025).

The indication-specific concordance rates provide additional descriptive context. These rates were based on exact agreement between the first-listed primary ICD-10 codes recorded at the referral, EC pre-test, and final EDX stages. They should not be interpreted as comparative measures of diagnostic accuracy because the subgroup sizes were small, the referral documentation was not standardized, and the EC pre-test and final classifications were made by the same physician.

An immediate management recommendation or intended plan modification was recorded in 53 of 101 patients (52.5%)The study did not verify whether the referring clinician accepted the recommendation, whether a proposed investigation, referral, procedure, or treatment was completed, or whether the patient experienced clinical benefit. The observed associations between diagnostic change, referral nonconfirmation, and recorded management modification indicate that EDX findings were associated with the consultant's immediate management recommendations, but they do not establish downstream effectiveness or improved outcomes.

This study has important limitations. The single-center design, modest sample size, and referral mix limit generalizability. Incorporation, observer, and expectation bias are major limitations because the same consultant performed the pre-test assessment and interpreted the final EDX evaluation. Referral diagnoses were abstracted from routine documentation and may not reflect the referring clinician's complete examination or differential diagnosis. Referral confirmation, diagnostic change, and recorded management-plan modification were classified by the EC and were not independently adjudicated, introducing additional potential for subjective classification bias. Finally, management-change types were not consistently coded in the analytic dataset; the binary management outcome should therefore be interpreted as an overall measure of recorded management modification.

These findings are hypothesis-generating. Larger multicenter studies should use standardized pre-test assessments, independent or blinded EDX interpretation, prespecified disease-specific analyses, independent adjudication of management changes, and patient-centered outcome measures.

## 5 Conclusion

In this single-center exploratory prospective cohort, a focused pre-test assessment by the electrodiagnostic consultant agreed with final EDX findings more frequently than did the referral diagnosis, but this comparison reflects within-clinician agreement and is vulnerable to incorporation and observer bias. Final EDX provided incremental diagnostic information after the focused pre-test assessment in 28.7% of cases and was frequently associated with an immediate management recommendation or intended plan modification. The study did not determine whether these recommendations were implemented or improved clinical outcomes. EDX should be considered a complement to a careful clinical assessment, not a replacement for it. Larger multicenter studies with independent or blinded assessments are needed.

## Funding

This research received no specific grant from any funding agency in the public, commercial, or not-for-profit sectors.

Ethics approval

This study was approved by the Institutional Review Board of ******. Verbal informed consent was obtained from all participants.

Data availability

De-identified patient data supporting the findings of this study are available from the corresponding author upon reasonable request. Data are not publicly available due to privacy and consent restrictions, as participants consented only for anonymous use within the study institution.

## Declaration of competing interest

The authors declare that they have no competing interests.
